# Measurement properties of patient-reported outcome measures used in rehabilitation of adults with chronic musculoskeletal pain: A mapping review

**DOI:** 10.3233/BMR-220133

**Published:** 2023-05-25

**Authors:** A.J.A. Köke, C.H.G. Bastiaenen, J. Kleijnen, I. Telgenkamp, R.J.E.M. Smeets, L.W.M.E. Beckers

**Affiliations:** a Department of Rehabilitation Medicine, Care and Public Health Research Institute (CAPHRI), Faculty of Health Medicine and Life Sciences, Maastricht University, Maastricht, The Netherlands; b Adelante Centre of Expertise in Rehabilitation and Audiology, Hoensbroek, The Netherlands; c Faculty Health and Technology, Zuyd University for Applied Sciences, Heerlen, The Netherlands; d Pain in Motion International Research Group (PiM), Brussels, Belgium; e Department of Epidemiology, Care and Public Health Research Institute (CAPHRI), Faculty of Health Medicine and Life Sciences, Maastricht University, Maastricht, The Netherlands; f Kleijnen Systematic Reviews Ltd, York, UK; g Centre for Integral Rehabilitation (CIR), Eindhoven, The Netherlands

**Keywords:** Validity, reliability, responsiveness, measurement tool, translation

## Abstract

**BACKGROUND:**

Choosing measurement tools for diagnostic, prognostic, or evaluative purposes in a chronic musculoskeletal pain (CMP) population is challenging for rehabilitation practice. Implementation of measurement tools for clinical practice is impaired by gaps in knowledge about measurement properties.

**OBJECTIVE:**

Identifying evidence about the measurement properties of tools frequently used in Dutch pain rehabilitation practice.

**METHODS:**

A mapping review was conducted of eligible studies that investigated *reliability*, *validity*, or *responsiveness*, and *interpretability*, as defined by the COSMIN taxonomy, of original versions or Dutch translations of predefined Patient-Reported Outcome Measures (PROMs) in a CMP population. MEDLINE, PsycINFO, EMBASE, and CINAHL were searched in March 2021. Results were visually mapped.

**RESULTS:**

Thirty-five studies were included. The results show many knowledge gaps in both original and translated versions. In general, aspects of validity were most frequently reported. The Pain Disability Index, Pain Catastrophizing Scale, and the 12-Item Short Form Health Survey were the most studied measurement tools. No results were found for the Checklist Individual Strength, Illness Perception Questionnaire, and Utrecht Coping List.

**CONCLUSION:**

Little evidence of the measurement properties of PROMs used in rehabilitation of patients with CMP in the Netherlands was found. PROMs need to be used and interpreted with caution in daily practice.

## 1. Introduction

Rehabilitation in the field of chronic musculoskeletal pain (CMP) is based theoretically on the biopsychosocial model [1]. The main aim is to enable the patient to deal better with pain and pain-related disabilities in order to improve daily functioning and participation. The selection of specific treatment modules is preceded by the assessment of relevant factors that maintain chronic pain and associated disabilities. Subsequently, when conducting a rehabilitation programme, proper measurement of factors maintaining pain and disability considered relevant, as well as of outcomes, is extremely important [1]. The purposes of such measurements can be diagnostic, including setting treatment goals (focusing on problems that need to be targeted to achieve the patient’s treatment goals), prognostic (predicting outcomes), or evaluative (evaluation of treatment goals) [2]. The measurement tools used could be a part of history taking, physical examination, neurophysiological testing, or imaging, and could include biomarker measurements, performance tests, or a wide array of patient-reported outcome measures (PROMs). PROMs are identified through self-completed questionnaires, with the goal of enabling patients to rate their own perceived physical, psychological and/or social functioning, participation, and/or quality of life [1, 3]. The use of PROMs, as part of the overall assessment, continues to expand beyond clinical research, in recognition of its potential for daily care by supporting patient-centred approaches and contributing to shared decision-making.

Choosing from the large variety of available PROMs, identifying the most appropriate measure(s) for predefined diagnostic, prognostic or, most relevant, evaluative purposes within the population being treated, is a real challenge for clinical practice [4]. In the field of pain rehabilitation, several attempts and initiatives have been described for developing a minimum set of tools. This set should be used in clinical practice to support informed clinical decision-making, for prognosis, to monitor treatment progress, to evaluate outcomes, and also to facilitate the comparison and pooling of data for scientific purposes. The Dutch Dataset Pain Rehabilitation (DDPR), in addition to others like the Initiative on Methods, Measurement, and Pain Assessment in Clinical Trials (IMMPACT) and Outcome Measures in Rheumatoid Arthritis Clinical Trial (OMERACT), is an example of such a set and has been chosen as the core outcome set of PROMs for clinical practices providing interdisciplinary multimodal pain programmes in the Netherlands [1]. The set has been implemented in part of Dutch chronic pain rehabilitation facilities but there are, however, important drawbacks to this implementation, such as the lack of a systematic overview of the research literature on the measurement properties of the tools. This also applies to PROMs that health care professionals use in addition to the DDPR. An important first step to aid further research and eventually for adopting tools in daily practice is an overview of which measurement properties have already been studied, and which have not.

The Consensus-based Standards for the selection of health Measurement Instruments (COSMIN) advocates a scientifically sound taxonomy, which groups measurement properties for health measurement tools, including PROMs, into the domains of *reliability*, *validity*, and *responsiveness*. The COSMIN taxonomy is a recommended resource in the identification of knowledge gaps in clinimetric evaluations of PROMs [5]. An additional characteristic is the *interpretability* of the outcome scores themselves. Within the taxonomy of the COSMIN, interpretability is considered the assignment of clinical or commonly understood connotations to a measure’s quantitative collected outcome scores or change in scores [2]. As such, interpretability cannot be considered a measurement property like reliability, validity, and responsiveness because it does not refer to the clinimetric quality of a measure itself. However, interpretability stands for the translation from quantitative scores into a clinically meaningful message to the patient and caregiver, which clearly demonstrates its great importance for clinical practice [2]. Unfortunately, interpretability often receives little attention in science or clinical practice and, therefore, may be an insufficiently studied aspect of the measurement tools, along with measurement properties [2].

As indicated above, a mapping review is a significant first step in increasing the evidence about and application of measurement tools. The goal of a mapping review is to identify and categorize existing literature to define research priorities and make informed decisions for systematic reviews as well as primary studies. The research field is described through a systematic search in a broad field and presentation of the results in a user-friendly format, often a visual figure or graph [6, 7, 8]. The scope for this mapping review is defined as follows:

1. To systematically map out existing literature about the measurement property domains *reliability*, *validity*, and *responsiveness* (as defined in the COSMIN taxonomy) for the tools proposed by the DDPR, expanded with additional frequently used tools, within pain rehabilitation clinical practice.
2. To systematically map out existing literature about the *interpretability* (as defined in the COSMIN taxonomy) of these tools.

## 2. Materials and methods

A mapping review was conducted to provide a broad overview of existing studies on measurement properties for commonly used PROMs in clinical pain rehabilitation programmes in the Netherlands.

### 2.1 Eligibility criteria

To be included, full-text articles and conference abstracts had to present original data. Any type of study that used PROMs only as outcome measurements was excluded, as these provide only indirect evidence about measurement properties of PROMs. No restriction was imposed as to publication date. The languages of the records were restricted to English, German, and Dutch. Inclusion criteria for PROMs, measurement properties, and the chronic pain population were specified as follows.

#### 2.1.1 PROMs

Eligible studies had to present data on measurement properties of at least one of the predefined PROMs commonly used in pain rehabilitation centres in the Netherlands: the Hospital Anxiety and Depression Scale (HADS), Pain Catastrophizing Scale (PCS), Pain Disability Index (PDI), and Patient Specific Complaint (PSC), all part of the DDPR, and the Checklist Individual Strength (CIS), Psychological Inflexibility in Pain Scale (PIPS), Illness Perception Questionnaire (IPQ), Pain Self-Efficacy Questionnaire (PSEQ), 12-Item Short Form Health Survey (SF-12), Symptom Checklist-90 (SCL-90), and Utrecht Coping List (UCL). The latter six PROMs are highly relevant for clinical practice in addition to the DDPR and were selected based on the clinical expertise of the research team. Derivatives of PROMs were included as well. Studies were included if the measurement properties of the original PROM were reported, as were studies in which the measurement properties of the Dutch version of an original PROM were reported.

An overview of the list of PROMs, their intended constructs, and their purpose is presented in Table 1 [9].

#### 2.1.2 Measurement properties

Eligible studies had to present data on at least one measurement property in the domains reliability, validity, responsiveness, or on interpretability [5, 9, 10]. Definitions by the COSMIN of the domains and the measurement properties thereof were used to assess eligibility [5].

The domain *reliability* comprises reliability, measurement error, including test-retest and absolute agreement, and internal consistency;

The domain *validity* includes content validity (i.e., face validity), criterion validity (i.e., concurrent and predictive validity), and construct validity (i.e., structural validity, cross-cultural validity, and hypotheses testing);

*Responsiveness* contains measurement properties that refer to the ability of an outcome measure to detect whether change over time in the construct being measured has indeed occurred, or not. Responsiveness is also considered longitudinal validity, but separately described in the COSMIN taxonomy for reasons of the timing of the measurement (validity is only cross-sectional while responsiveness makes use of two measurements over time);

*Interpretability* represents the degree to which one can assign qualitative meaning – that is, clinically or commonly understood connotations – to a PROM’s quantitative scores or change in scores. Several aspects can be used to provide additional quantitative information about interpretability: distribution scores, floor and ceiling effects, and minimally important change values.

#### 2.1.3 Population

Studies were only included if they reported data on measurement properties of listed PROMs in a population with chronic primary pain (e.g., chronic primary musculoskeletal pain and chronic widespread pain (e.g., fibromyalgia)), chronic secondary pain (e.g., chronic post-surgical or post-traumatic pain (e.g., chronic pain whiplash injury)), chronic secondary musculoskeletal pain (e.g., osteoarthritis), or chronic neuropathic pain, according to the International Classification of Diseases ICD-11 classification of chronic pain [11]. Studies with a population of patients with chronic pain aged 16 years or older were included, since, as of 2018, the age of 16 is considered legal for independent medical-related decision-making in the Netherlands. Studies including mixed populations, other than chronic pain, were only included if the results were reported separately for the eligible population.

### 2.2 Data sources and searches

Multiple electronic data sources were searched in

**Table 1:** Construct and purpose (i.e., discriminative (D), evaluative (E), and predictive (P)) depicted for the original developed PROM version and the translated Dutch version

PROM	Language	First author	Year	Construct	Purpose PROM		
					D	E	P
HADS	English${}^{†}$	Zigmond [12]	1983	“To detect anxiety and depression among patients in medical settings.”	x	x	
	Dutch	Spinhoven [13]	1997	“To provide a screening measure for the presence of anxiety and depression specifically and not for a global psychiatric disorder in general.”	x		
PCS	English${}^{†}$	Sullivan [14]	1995	“To reflect on past painful experiences and to indicate the degree to which they experienced each of 13 thoughts or feelings when experiencing pain.”	x	x	x
	Dutch	Crombez [15]	1996	“To measure the extent of catastrophizing within pain.”	x	x	
PDI	English${}^{†}$	Pollard [16]	1984	“To measure the extent to which chronic pain interferes with a person’s disability to engage in various life activities.”	x	x	
	Dutch	Soer [17]	2013	“To measure and evaluate disability associated with pain.”	x	x	
PSC	Dutch${}^{†}$	Beurskens [18]	1999	“To assess functional status in patients with low back pain.”		x	
CIS	Dutch${}^{†}$	Vercoulen [19]	1994	“To measure several aspects of fatigue.”	x	x	x
PIPS	Swedish${}^{†}$	Wicksell [20]	2008	“To assess relevant aspects of psychological in/flexibility, such as avoidance and cognitive fusion.”	x	x	x
	Dutch	Trompetter [21]	2014	“To measure psychological inflexibility.”			
IPQ	English${}^{†}$	Weinman [22]	1996	“To assess cognitive representations of illness.”	x	x	x
	Dutch	de Raaij [23]	2012	“To assess illness perceptions.”	x	x	
PSEQ	English${}^{†}$	Nicholas [24]	2007	“To take pain into account when rating their self-efficacy beliefs.”	x	x	x
	Dutch	van der Maas [25]	2012	“To assess participants’ confidence in their ability to perform activities of daily living despite the pain.”	x	x	x
SF-12	English${}^{†}$	Ware [26]	1996	“To measure health status.”	x	x	
SF-12	Dutch	Mols [27]	2009	“To compare health status between groups of patients, to identify predictors of health status.”	x	x	
SCL-90	English${}^{†}$	Derogatis [28]	1973	“To rate multidimensional symptom distress.”	x	x	
SCL-90	Dutch	Arrindell [29]	2003	“To measure psychological distress.”${}^{*}$	x	x	
UCL	Dutch${}^{†}$	Schreurs [30]	1984	“To measure coping strategies in problematic or adjustment demanding situations.”	x		

${}^{†}$Language of original developed PROM. ${}^{*}$Interpretation of the reviewers based on ambiguous information. Abbreviations: CIS: Checklist Individual Strength; HADS: Hospital Anxiety and Depression Scale; IPQ: Illness Perception Questionnaire; PCS: Pain Catastrophizing Scale; PDI: Pain Disability Index; PIPS: Psychological Inflexibility in Pain Scale; PSEQ: Pain Self-Efficacy Questionnaire; PSFS: Patient-Specific Functional Scale; SCL-90: Symptom Checklist-90, SF-12: 12-Item Short Form Health Survey; UCL: Utrecht Coping List.

March 2021: MEDLINE, including in-process and other non-indexed citations, Epub ahead of print, and daily update (Ovid), PsycINFO (Ovid), EMBASE (Ovid), and CINAHL (EBSCOhost). Specific search queries encompassed both controlled vocabulary (e.g., MESH terms) and text words in titles and abstracts. The search strategies, tailored for each electronic data source (*Supplement 1*), were developed by author IT and peer-reviewed by JK. An information specialist of Kleijnen Systematic Reviews Ltd conducted the searches in all databases. Potentially relevant records were collected and duplicates were removed, both using EndNote X9 and manually, using the web-based system Rayyan (based on publication year, journal, volume, and issue) [31]. Included records, excluded literature reviews, and reviewers’ personal libraries were screened for relevant records for inclusion as well.

### 2.3 Study selection

During the first phase, two initial reviewers, IT and either AK, RS, or LB, independently screened the search results for eligible studies on title and abstract, using Rayyan. During the second phase, the same initial reviewers screened the selected records in duplicate based on full-text articles according to the predefined eligibility criteria Disagreements regarding the inclusion of specific records during both phases were discussed by the two initial reviewers and, if necessary, mediated by a third reviewer.

### 2.4 Data charting

Specifics on included studies were charted: author(s), publication year, PROM of interest, language of the investigated PROM, setting in which the study was conducted, country, the population of interest, and the study sample’s age, gender, and pain duration. In addition, an overview of studies for each measurement domain and its specific properties was presented in a table and additionally visually mapped. Data extraction for measurement properties was performed as described in the original studies, according to COSMIN taxonomy [5], by one author (IT). In the case of ambiguous reporting, allocation of measurement properties was performed by author IT. A second author (LB) randomly verified 20% of the data extraction. As mapping reviews focus on the quantity and key characteristics of literature, quality assessment of included studies and/or methodologies is not indicated and thus no part of this study.

## 3. Results

**Figure 1.**
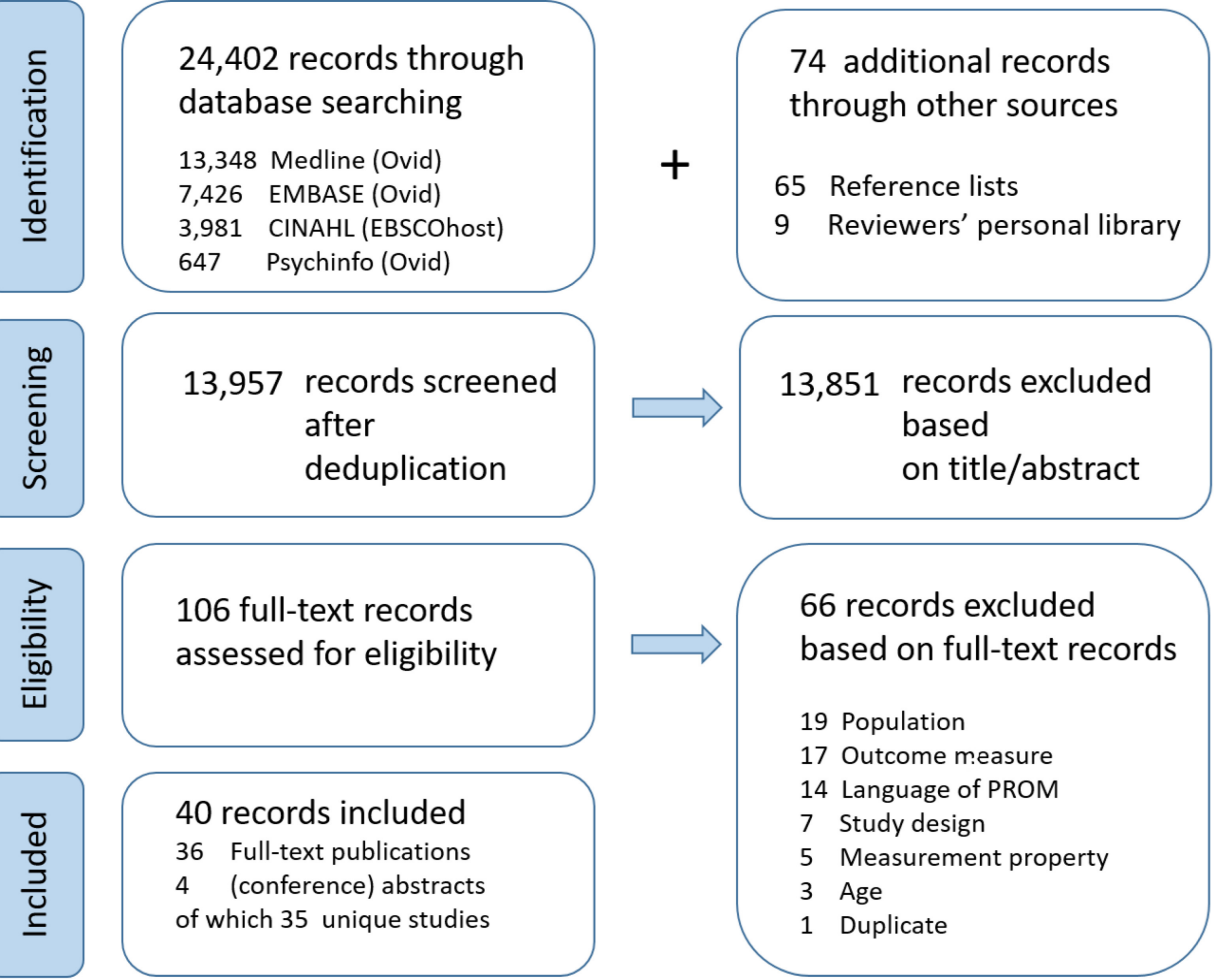
The flowchart representing the identification, screening, and eligibility process.

A total of 24,476 records were identified, of which 24,402 were obtained through database searching and 74 through other sources (reference tracking and reviewers’ personal libraries). After deduplication, 13,957 records were screened based on title and abstract (Fig. 1). Consequently, 106 full-text records were assessed for eligibility, of which 40 were included in the quantitative synthesis. Thirty-six full-text publications and four (conference) abstracts were distinguished. Finally, 35 unique studies were identified for inclusion.

The study populations of the majority of studies consisted of groups of patients with different diagnoses: chronic primary musculoskeletal pain (e.g., low back, neck, shoulder, and knee pain), chronic widespread pain (e.g., fibromyalgia), chronic post-traumatic pain (e.g., whiplash injury), and/or chronic secondary musculoskeletal pain (e.g., arthritis). In addition, studies were conducted in different settings (hospitals, rehabilitation centres, pain centres, primary care, or national surveys), or included patients from several settings. The mean reported duration of pain varied from 3 to 162 months. The average age of all populations was 46.5 years (SD 6.4, range 32.1–68.6).

**Table 2:** Overview included studies per PROM

	Original language	(E/S/D)	Dutch translation
HADS	2	E	1
PCS	4${}^{\$}$	E	3${}^{\$}$
PDI	10${}^{*}$	E	4${}^{*}$
PSC	2	D	
CIS	0	D	
PIPS	2	S	1
IPQ	0	E	0
PSEQ	5	E	1
SF-12	3	E	0
SCL-90	4	E	0
UCL	0	D	

Abbreviations: CIS: Checklist Individual Strength, HADS: Hospital Anxiety and Depression Scale; IPQ: Illness Perception Questionnaire; PCS: Pain Catastrophizing Scale; PDI: Pain Disability Index; PIPS: Psychological Inflexibility in Pain Scale; PSEQ: Pain Self-Efficacy Questionnaire; SCL-90: Symptom Checklist-90; SF-12: 12-Item Short Form Health Survey; UCL: Utrecht Coping List. E = English, S = Swedish, D = Dutch. ${}^{\$}$Including Delphi-study. ${}^{*}$Including study described results of Dutch and English version together.

Results for measurement properties were found for the HADS, PCS, PDI, PSC, PIPS, PSEQ (including a short form PSEQ-2 item version), SF-12 (including a derivative SF6D12), and SCL-90 (including SCL-90-R). No studies, in chronic pain populations, were found for the CIS, IPQ, and UCL.

Thirty-one studies included PROMs in the language of original development (English (n= 27), Dutch (n= 2), Swedish (n= 2)). In addition, one study evaluated a PROM (PDI) in both the original (English) and translated versions (Dutch), and eight studies investigated PROMs translated into Dutch. One included article reported the results of a Delphi study for consensus on the most appropriate PROM to assess psychosocial risk factors (PSEQ and PCS) in patients with chronic pain. These results were regarded as an outcome for the domain of validity (content validity). Four articles included results for two PROMs: HADS/PDI, PCS/PSEQ, SF-12/PDI, and SF-12/SCL-90.

The numbers of included studies for each PROM in the original version and translated version (Dutch) are presented in Table 2. The study characteristics of the included records for each PROM are presented in Supplement 2.

### 3.1 Overview of measurement properties for original PROMs

**Figure 2.**
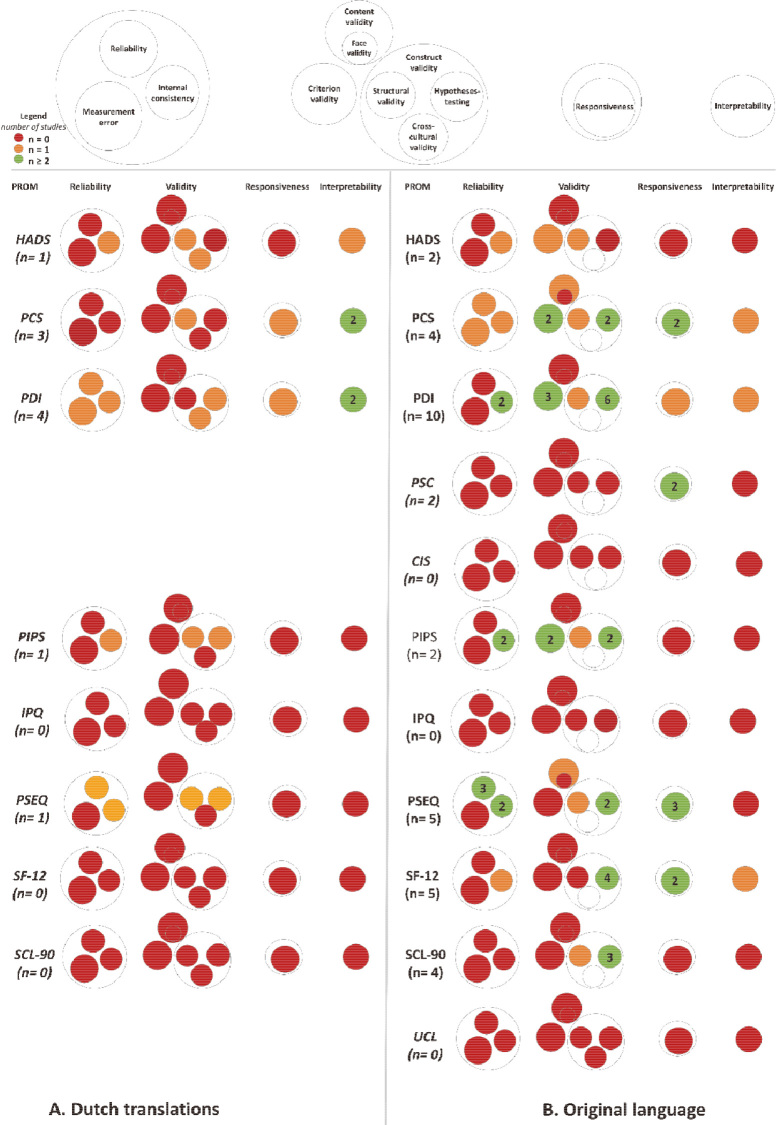
Data on measurement properties mapped in ascending order of the number of studies presented for each PROM (PROMs in bold and italicized = Dutch, bold = English, normal font = Swedish). The shapes of the COSMIN domains are according to the taxonomy developed by COSMIN [5]. Red indicates no studies, orange one study, and green two or more studies, blank does not apply (cross-cultural validation). Abbreviations: HADS: Hospital Anxiety and Depression Scale; PCS: Pain Catastrophizing Scale; PDI: Pain Disability Index; PSC: Patient Specific Complaints; CIS: Checklist Individual Strength; PIPS: Psychological Inflexibility in Pain Scale; IPQ: Illness Perception Questionnaire; PSEQ: Pain Self-Efficacy Questionnaire; SF-12: 12-Item Short Form Health Survey; SCL-90: Symptom Checklist-90; UCL: Utrecht Coping List.

The data, as mapped in Fig. 2 and presented in Table 3, show several knowledge gaps for the original PROMs in the population of patients with CMP. Out of all the original language PROMs included, the PCS had the most measurement properties studied. Studies have been carried out for all COSMIN domains and every measurement property included in these, apart from face validity. Likewise, most measurement properties of the original PDI were studied, again covering all COSMIN domains, apart from reliability and measurement error in the domain reliability and face validity in the domain validity. Despite the fact that studies reported results within all domains of the SF-12, results for reliability, measurement error, content and criterion validity, as well as structural validity, were lacking. For the PSEQ, no studies at all reported on interpretability, while, in the domains reliability and validity, there were several knowledge gaps. No studies reported on responsiveness and interpretability for the HADS and PIPS and there were gaps for both in the domains reliability and validity. For the SCL-90, only results for the domain validity were found. For the original Dutch PSC, only responsiveness was studied. Furthermore, no studies at all were found for the CIS, IPQ, and UCL.

### 3.2 Overview of measurement properties of translated PROMs 

Of the PROMs translated into Dutch, cross-cultural validity was only reported on for the HADS and PDI. Most measurement properties were studied for the Dutch PDI, with the exception of face, criterion and structural validities. For the PCS, no studies were found for the domain reliability, and in the domain of validity, only structural validity was examined. In the domain reliability, internal consistency was tested for HADS, PIPS, and PSEQ, with reliability as well for the PSEQ. In the domain validity, structural validity was reported for all three and hypotheses testing for the PSEQ and PIPS. Only interpretability was studied for HADS, but

**Table 3:** Investigated measurement properties presented in included studies for each PROM

	PROM	Author	Year	Reliability	Internal consistancy	Measurement error	Content validity	Criterion validity	Structural validity	Hypotheses testing	Cross cultural validaty	Responsiveness	Interpretability	Other
Dutch	HADS	Giusti [32]	2020		√				√CFA		√		√	√EFA
language	PCS	van Damme [33]	2000										√RV	
PROMs	PCS	van Damme [34]	2002						√CFA					
	PCS	Pulles [35]	2020									√	√	
	PDI	Soer [36, 37]	2011,									√	√	
			2012											
	PDI	Soer [17]	2013	√	√	√				√	√			
	PDI	Soer [38]	2015										√	
	PSC${}^{*}$	Beurskens [39]	1996									√		
	PSC	Beurskens [18]	1999									√		√FE
	PIPS	Trompetter [21]	2014		√				√CFA	√				√EFA${}^{*}$
	PSEQ	van der Maas [25]	2012	√	√				√CFA	√				√EFA${}^{*}$
Non-dutch	HADS	Pallant [40]	2005		√				√CFA					√EFA
language	HADS	Rusu [41]	2016					√CV						
PROMs	PCS	Osman [42]	2000		√			√CV	√CFA	√			√RV	
	PCS	George [43]	2010	√		√		√CV${}^{*}$				√		
	PCS	Prime [44]	2012							$\surd^{*}$				
	PCS	Sleijser [45]	2019				√							
	PDI	Pollard [16]	1984							√				
	PDI	Tait [46]	1987		√				$\surd^{*}$CFA					
	PDI	Jerome [47]	1991							√				
	PDI	Millard [48]	1991							√				
	PDI	Strong [49]	1994		√			√CV		√				
	PDI	Crighton [50]	2014					√CV		√				
	PDI	Soer [38]	2015										√RV	
	PDI	Morris [51]	2015									√		
	PDI	Rusu [41]	2016					√CV						
	PDI	McKillop [52]	2018							√				
	PIPS	Wicksell [20]	2008		√			√CV		√				√EFA${}^{*}$
	PIPS	Wicksell [53]	2010		√			√CV	√CFA	√				√EFA${}^{*}$
	PSEQ	Nicholas [24]	2007	√	√					√				
	PSEQ	Maughan [54]	2010								√			
	PSEQ	Nicholas${}^{**}$ [55]	2015	√	√				√CFA	√		√		√EFA
	PSEQ	Costa [56]	2017	√										
	PSEQ	Sleijser [45]	2019				√							
	SF-12	Luo${}^{**}$ [57]	2012		√					√		√	√∗	
	SF-12	Morris [51]	2015									√		
	SF-12	Tawiah [58]	2018							√				
	SF-12	Tawiah [59]	2019							√				
	SF-12	Kroenke${}^{**}$ [60]	2019							√		√		
	SCL-90-R	Kinney [61]	1991							√				
	SCL-90-R	Bernstein [62]	1994						√CFA	√				
	SCL-90-R	Peebles [63]	2001							√				
	SCL-90	Kroenke${}^{**}$ [64]	2019							√		√		

${}^{*}$Interpretation of the reviewers based on ambiguous information. Abbreviations: HADS: Hospital Anxiety and Depression Scale; PCS: Pain Catastrophizing Scale; PDI: Pain Disability Index; PIPS: Psychological Inflexibility in Pain Scale; PSEQ: Pain Self-Efficacy Questionnaire; PSC: Patient Specific Complaints; SCL-90: Symptom Checklist-90; SF-12: 12-Item Short Form Health Survey; CV: Concurrent Validity; RV: reference values; FE: feasibility; CFA: Confirmatory Factor Analysis; EFA: Explorative Factor Analysis. ${}^{**}$study of derivative or subscale of original PROM.

not responsiveness; for PIPS and PSEQ, responsiveness and interpretability were not studied. No studies on the Dutch SF-12 and SCL-90 were found in populations of patients with chronic pain.

## 4. Discussion

The objective of this study was to map out the literature available on the reliability, validity, responsiveness, and interpretability of PROMs commonly used in Dutch pain rehabilitation, including measurement tools proposed by the DDPR. The results show many knowledge gaps, although more studies were found for the PROMs in their original languages than for translated versions. Information has been published for all domains, but not all measurement properties, of both the original and Dutch version of the PDI and the original versions of the PCS and SF-12. For the original Dutch CIS, IPQ, and UCL, as well as for the Dutch translations of the SF-12 and SCL-90, no information was found on any measurement property. Overall, for the other tools, aspects of validity were most frequently reported, followed by reliability, responsiveness, and interpretability.

The results of this study complement a previous evidence review for a population with CMP evaluating measurement properties of PROMs for pain severity and pain-related functional impairment not included in the current review. For only three of the 13 multi-item tools (Oswestry Disability Index (ODI); Roland-Morris Disability Questionnaire (RMDQ); SF-36 Bodily Pain Scale (SF-36 BPS)), were data found for all COSMIN domains [65]. Interestingly, the PROMs investigated in the present study had already been developed in the ’70s, ’80s, or ’90s, apart from the PSEQ (2007) and PIPS (2008). Since their development, PROMs have been widely used both clinically and in research, and are nowadays recommended as core outcome measures by the DDPR. The limited number of clinimetric studies performed in the CMP population in recent decades, despite the prominent role of these questionnaires, clearly points out a shortcoming in prior and current practices. At the same time, the paucity of research found by this review suggests that both researchers and funders have insufficiently acknowledged the importance of clinimetric research. An additional illustration of this point is the fact that a selective group of researchers is responsible for the majority of studies concerning specific PROMs, such as the PDI, which is unfavourable considering research biases. 

The finding that responsiveness has received less attention than reliability and validity may be explained by the challenges in executing and interpreting research on this, including the necessity of multiple repeated measurements, as well as the fact that not all PROMs have an evaluative purpose. Interpretability, of high importance as it gives clinical meaning to (change in) scores, has been unjustifiably ignored. The importance of interpretation of scores was underlined during the implementation of the DDPR, as the lack of information on interpretability was a frequently mentioned reason for the PROMs not being feasible or relevant to use in practice [66]. It is also striking that only a single study reported on the content validity of a measure, while COSMIN considers it the most important measurement property. This is because the content of a measurement instrument should be relevant, comprehensive, and comprehensible with respect to the construct of interest and to the target population [5].

A major strength of this review is that it was performed completely within the theoretical framework of the COSMIN taxonomy, which is based on international consensus [5]. However, as the extraction of measurement properties was determined by the authors’ reporting instead of reviewer assessment, the allocation may not entirely be in accordance with the taxonomy’s definition. This limitation is a particular concern for PROMs developed before the publication of the taxonomy, and where the terminology used in a study is ambiguous. Content validity and pilot testing (not included in this review), for example, can involve overlapping aspects, and factor analysis can be part of both construct validity and field testing (not included) [2]. Similarly, potentially relevant studies may not have been identified due to discrepancies between the terms in our comprehensive search strategy and the terminology of measurement properties used by authors of such studies, resulting in selection bias. This may also explain the finding that nine studies from reviewers’ personal libraries were not identified by our search strategy.

Another strength was the broad range of PROMs examined, covering many aspects of the biopsychosocial model. They were chosen pragmatically, emerging from the DDPR and the clinical experience of the research team. Nevertheless, a limitation of the selection’s being made by a narrow group is that other relevant questionnaires and corresponding studies may have been missed. The Global Perceived Effect (GPE), Numerical Rating Scale (NRS), and Visual Analogue Scale (VAS), although part of the DDPR, were considered nonspecific response scales: they are used to measure a variety of constructs and, correspondingly, adopt diverse question wordings, anchors at either end of the scale, time spans, and presentations. Given the limited comparability of the different variants, single-item GPE, NRS, and VAS scales were not included in this review. Last, the objective was restricted to the language of the original PROMs and their Dutch translations. Therefore, it is not possible to generalize our results to other languages and cultures, although similar trends, or even fewer studies, could be expected. 

For all PROMs, more research is needed to fill the knowledge gaps about their measurement properties, with particular attention to content validity and interpretability. At the same time, for the Dutch versions of the PDI, PCS, and PSC as well as for the original language versions of the PDI, PCS, PSEQ, PIPS, SF-12, and SCL-90, sufficient data seem available to perform systematic reviews to describe their measurement properties, synthesize the data, and perform quality assessments of studies. Specific attention is required as to whether the measurement properties of investigated tools are in line with their intended purposes (diagnostic, predictive, evaluative). The focus of further work could be extended to translations other than into Dutch and to other PROMs. Several research teams performing (replication) studies would likely produce more valid and generalizable conclusions. While the COSMIN taxonomy and the other COSMIN tools are recommended for future studies, their applicability or otherwise to the specific field of rehabilitation medicine should be considered. For instance, the lack of confirmed clinimetrically sound comparator measurement instruments can make testing hypotheses imprecise. Moreover, adequate sample sizes, as indicated in the COSMIN study design checklist, can be challenging to accomplish within the setting of CMP, especially if one aims to examine measurement properties in subpopulations.

While awaiting the results of future clinimetric studies, researchers need to be critical when using PROMs and transparently acknowledge any limitations. Policy- and decision-makers, including health insurance companies, should not overestimate the impact of either individual or group-based PROM outcomes and be careful when drawing conclusions from them. Likewise, health care professionals in rehabilitation should be aware of the limited evidence of PROMs’ clinimetric qualities. Given the heterogeneity of the CMP population and the complexity of rehabilitation interventions, it is in any case of the utmost importance to guide the rehabilitation trajectory, based on combinations of measurement instruments within the biopsychosocial model, together with the clinical expertise of the interdisciplinary team and the patient’s perspective. However, this necessity to integrate outcomes of multiple PROMs covering the total biopsychosocial spectrum when assessing CMP patients, can lead to extensive sets of questionnaires to be completed by the patient, not only in the diagnostic phase but also during and after treatment.

Given the associated burden of this for patients, item banks and computer adaptive testing (such as the Patient-Reported Outcomes Measurement Information System (PROMIS)) that have been validated could be considered as alternatives to traditional patient-reported outcome measurements. For the sake of comparability, a collective approach based on expert consensus between representatives of health care professionals, researchers, and patients is desirable, and an important focus for future work.

## 5. Conclusion

The studies included in this mapping review demonstrate a paucity of evidence for the reliability, validity, responsiveness, and interpretability of most PROMs frequently used in rehabilitation of patients with CMP in the Netherlands. The main implication is that these PROMs need to be used and interpreted with caution in daily practice.

## Ethical approval

Not applicable.

## Funding

This work was supported by the Centre for Integral Rehabilitation (CIR). The public partner, Maastricht University, is responsible for the study design, data collection and analysis, decision to publish, and preparation of the manuscript.

## Informed consent

Not applicable.

## Author contributions

Study design and conceptualizations: S.R., B.L., Search strategy: K.J., I.T., B.L., Databases searching: I.T. B.L., data screening K.A., I.T., S.R., B.L., Data extraction: K.A., I.T., B.L., Data synthesis and interpretation: K.A., B.C.,S.R., B.L., Manuscript drafting B.L., Writing review K.A. B.C., K.J., S.R., B.L., Editing review and visualization K.A., Supervision S.R., B.L. All authors read and approved the final version of the manuscript.

## Supplementary data

The supplementary files are available to download from http://dx.doi.org/10.3233/BMR-220133.

## Supplementary Material

Supplementary
