# Supplementary material for: Measurement properties of patient-reported outcome measures used in rehabilitation of adults with chronic musculoskeletal pain: A mapping review
Source: J Back Musculoskelet Rehabil. 2023 May 25;36(3):595–607. doi: 10.3233/BMR-220133 (PMC10200236; doi:10.3233/BMR-220133)
Supplement: Supplementary [file bmr-36-bmr220133-s001.docx]

**Supplement 1 Example of search strategy (MEDLINE (Ovid))**

1 ((chronic* or constant* or continu* or deep-rooted or deep-seated or dire or grave or habitual or incessant or incurable or immedicable or ineradicable or intractable or lingering or lasting or long* or persist* or permanent or protracted or perennial or root* or recurr* or severe or serious* or sustain* or unabating or unceasing or unending or unrelenting or unremitting) adj3 (ache* or aching or affliction or agony or cramp* or discomfort or excruti* or hurt* or irritation or pain* or pang* or prickl* or rack* or smart* or sore* or spasm* or stab* or stiffness or sting* or stitch* or strain* or suffer* or tender* or throb* or tingl* or trauma or twing*)).ti,ab,ot.

2 exp chronic pain/ or Pain, intractable/

3 or/1-2

4 (body or bodily or musc* or skelet* or bone* or joint* or msp).ti,ab,ot.

5 exp musculoskeletal pain/ or exp muscles/

6 or/4-5

7 3 and 6

8 (PRO or PROM or PROMS or PREM or PREMS).ti,ab,ot.

9 (Patient report* adj2 (outcome* or assessment* or symptom* or satisfaction or satisfied or experience* or question*)).ti,ab,ot.

10 (Patient* adj2 (outcome assess* or outcome measure* or outcome tool* or outcome instrument* or outcome questionnaire* or outcome survey* or outcome score* or outcome scale* or process assess* or self-report* or self-assess* or satisfaction measure* or satisfaction tool* or satisfaction assess* or satisfaction instrument* or satisfaction questionnaire* or satisfaction survey* or satisfaction score* or satisfaction scale* or question sheet*)).ti,ab,ot.

11 ((healthcare or health care) adj (survey* or questionnaire* or form* or assess* or measure* or score*)).ti,ab,ot.

12 (Patient preference* adj2 (measure* or tool* or assess* or instrument* or questionnaire* or survey* or score* or scale*)).ti,ab,ot.

13 (NRS or numeric rating scale* or PDI or Disability index or PSFS or patient specific functional scale* or PCS or pain catastrophi?ing or measure* of pain catastrophi?ing or HADS or HADS-NL or "Hospital anxiety and depression scale*" or SF12 or Short form or "12 item*" or IPQ or Illness perception questionnaire* or PSEQ or Pain self efficacy questionnaire* or PIPS or Psychological inflexibility in pain scale* or SCL-90 or symptom checklist* or UCL or Utrecht coping list* coping strategies questionnaire* or psychological factors questionnaire* CIS or Checklist individual Strength*).ti,ab,ot.

14 exp Surveys/ and Questionnaires/

15 exp Psychological Tests/ or exp Health Surveys/ or exp Health Care Surveys/ or exp Symptom Assessment/ or exp Patient Outcome Assessment/ or exp Pain Measurement/ or exp Activities of Daily Living/ or exp Disability Evaluation/ or exp Self Concept/ or exp Recovery of Function/

16 or/8-15

17 (instrumentation or methods).fs. or (Validation Studies or Comparative Study).pt. or exp Psychometrics/ or psychometr*.ti,ab. or (clinimetr* or clinometr*).tw. or exp Observer Variation/ or observer variation.ti,ab. or exp Health Status Indicators/ or exp Reproducibility of Results/ or reproducib*.ti,ab. or exp Discriminant Analysis/ or (reliab* or unreliab* or valid* or coefficient or homogeneity or homogeneous or internal consistency).ti,ab. or (cronbach* and (alpha or alphas)).ti,ab. or (item and (correlation* or selection* or reduction*)).ti,ab. or (agreement or precision or imprecision or precise values or test-retest).ti,ab. or (test and retest).ti,ab. or (reliab* and (test or retest)).ti,ab. or (stability or interrater or inter-rater or intrarater or intra-rater or intertester or inter-tester or intratester or intra-tester or interobserver or inter-observer or intraobserver or intraobserver or intertechnician or inter-technician or intratechnician or intra-technician or interexaminer or inter-examiner or intraexaminer or intra-examiner or interassay or interassay or intraassay or intra-assay or interindividual or inter-individual or intraindividual or intra-individual or interparticipant or inter-participant or intraparticipant or intra-participant or kappa or kappas or kappas or repeatab*).ti,ab. or ((replicab* or repeated) and (measure or measures or findings or result or results or test or tests)).ti,ab. or (generaliza* or generalisa* or concordance).ti,ab. or (intraclass and correlation*).ti,ab. or (discriminative or known group or factor analysis or factor analyses or dimension* or subscale*).ti,ab. or (multitrait and scaling and (analysis or analyses)).ti,ab. or (item discriminant or interscale correlation* or error or errors or individual variability).ti,ab. or (variability and (analysis or values)).ti,ab. or (uncertainty and (measurement or measuring)).ti,ab. or (standard error adj2 (measurement or sensitiv* or responsive*)).ti,ab. or ((minimal or minimally or clinical or clinically) and (important or significant or detectable) and (change or difference)).ti,ab. or (small* and (real or detectable) and (change or difference)).ti,ab. or (meaningful change or ceiling effect or floor effect or Item response model or IRT or Rasch or Differential item functioning or DIF or computer adaptive testing or item bank or cross-cultural equivalence).ti,ab.

18 7 and 16 and 17

**Supplement 2 Study characteristics presented for the included study, the investigated PROM and target population**

| **PROM** | **Language of PROM** | **Author** | **Year** | **Country** | **Setting** | **N** | **Age (y) mean (SD)** | **Gender  Female N, %** | **Population  N, %** | **Pain duration mean (SD) or**  **frequencies** |
| --- | --- | --- | --- | --- | --- | --- | --- | --- | --- | --- |
| HADS | English* | Pallant [40] | 2005 | Australia | Rehabilitation center | 296 | 44.3 (12.47) | 152, 51.4% | CPMP | NR |
| HADS *(PDI)* | English* | Rusu [41] | 2016 | United Kingdom | Primary Care  Pain clinics | 78 | 45.26 (13.39) | 55, 70.5%* | CPMP | 78.54mo (93.11) |
| HADS | Dutch | Giusti [32] | 2020 | The Netherlands | Rehabilitation center  2 groups | total 10617* 1 2522 2 8095 | total NR  1 43.6 (12.5) 2 45.6 (12.5) | 1 1920, 76.2% 2 6116, 75.6% | 1 CPMP 2 NR | 1 16.5% < 1 y, 44.4% 1-5y, 38.9% >5 y  2 NR |
|  | | | | | | | | | | |
| PCS | English | Osman [42] | 2000 | United States of America | Two rehabilitation centers) | 60 | total NR | 34, 57%* | CPMP | NR |
| PCS | English | George [43] | 2010 | United States of America | University-based outpatient physical therapy clinics | 53 | 44.3 (18.5) | 43, 80% | CPMP | > 3mo |
| PCS | English* | Prime [44] | 2012 | Canada* | NR | 96 | NR | NR | CPMP | NR |
| PCS *(PSEQ)* | English* | Sleijser [45] | 2019 | Delphi study Europe North America | NR | 36 experts for consensus process | NA | NA | NA | NA |
| PCS | Dutch | van Damme [33; 34] | 2000, 2002 | Belgium, The Netherlands | NR  2 groups | 262 | total 42.7 (10.8)  1 41.5 (11.6) 2 44.6 (9.1) | total 179, 68%*   1. 99, 61%*   2 80, 80%* | 1 CPMP 162, 18%* 2 CWP 100, 82%* | NR |
| PCS | Dutch | Pulles [35] | 2020 | The Netherlands | Hospital  Rehabilitation center | 359 | 45.28 (10.97) | 238, 66% | CPMP | 47.6% < 5y  48.2% > 5y  4.2% N |
|  | | | | | | | | | | |
| PDI | English* | Pollard [16] | 1984 | United States of America* | Hospital* | 18 | NR | 6, 37.5%* | CPTP 9, 50%*  CPMP 9, 50%* | ≥ 6mo |
| PDI | English* | Tait [46] | 1987 | United States of America* | Hospital* Study 1  Study 2, 2groups | 108  73 | S1 44.6 (13.9)  S2  2a 38.6 (NR)  2b 45.6 (NR) | S1 49,45.37%*  S2  2a 31, 86.11%*  2b 19, 51,35%* | S1 CPMP  S2 NR | S1 6.8y (9.29)  S2 NR |
| PDI | English* | Jerome [47] | 1991 | United States of America* | Hospital | 74 | 46 (13.9) | 23, 31%* | CPMP | 4.1y (7.2) |
| PDI | English | Millard [48] | 1991 | United States of America | Hospital | 93 | 44.3 (11.2) | 54, 58% | CPMP | 51.2mo (73.6) |
| PDI | English | Strong [49] | 1994 | Australia | Hospital* | 100 | 46.3 (13.4) | 50, 50%* | CPMP | 9.7y (10.1) |
| PDI | Dutch | Soer [36; 37] | 2011,2012 | The Netherlands | University hospital Rehabilitation Center | 242 | 51 (NR) | 128* 53% | CPMP | NR |
| PDI | Dutch | Soer [17] | 2013 | The Netherlands | University hospital Rehabilitation Center  2 groups | 790 | total NR  1 48.8 (15.4)  2 43.6 (11.4) | total 572, 72%*  1 205, 48%  2 267, 73% | 1 CPMP 425, 53.8%  2 CWP 365, 46.2% | 1 CPMP 10.3y (10.2)  2 CWP 49% < 5y  51% > 5y |
| PDI | English* | Crighton [50] | 2014 | United States of America* | NR | 167 | 51.2 (14.2) | 94, 56.4%* | CPMP 147, 88%* Other NR 20, 12%* | NR |
| PDI *(SF-12)* | English* | Morris [51] | 2015 | NR | NR | 5965* | NR | NR | CPMP | NR |
| PDI | Dutch & English* | Soer [38] | 2015 | Canada The Netherlands | University hospital Rehabilitation center Provincial compensation database | 6997 | 49.3 (15.3) | 3778*, 54% | CPMP 4857, 69.4%* CWP 666, 9.5%* CPTP 689, 9.8%*  CNP 785, 11.2%* | NR |
| PDI  *(HADS)* | English* | Rusu [41] | 2016 | United Kingdom | Primary Care  Pain clinics | 78 | 45.26 (13.39) | 55, 70.5%* | CPMP | 78.54mo (93.11) |
| PDI | English | McKillop [52] | 2018 | Canada | Hospital | 70 | 48.1 (12.9) | 44, 62.9% | CPMP | 13.5y (9.7) |
|  | | | | | | | | | | |
| PSC | Dutch* | Beurskens [39] | 1996 | The Netherlands | Primary care | 81 | 41 (10) | 37, 46%* | CPMP | 70wks (119) |
| PSC | Dutch* | Beurskens [18] | 1999 | The Netherlands | Primary care | 150 | 41 (NR) | 66, 44%* | CPMP | NR |
|  | | | | | | | | | | |
| PSEQ | English | Nicholas [24] | 2007 | Australia | Hospital study1 Pain centre study2 Pain centre study3 | S1 103 S2 1306  S3 145 | S1 42 (NR) S2 41 (NR) S3 50.3 (13.25) | S1 53, 51.5%* S2 692, 53%* S3 86, 59%* | S1 CPMP S2 CPMP  S3 CPMP | S1 36mo (NR) S2 median 36mo  S3 9.9y (8.9) |
| PSEQ | English | Maughan [54] | 2010 | United Kingdom | Hospital | 63 | 52 (NR) | 32, 67% | CPMP | 6y (NR) |
| PSEQ 2 item version | English* | Nicholas [55] | 2015 | Australia | Pain centre  3 groups | total 1558  1 678  2 740  3 140 | 1 48 (15.6) 2 49.9 (16.1) 3 42.79(11.57) | 1+2 730, 57%*  3 72, 55%* | 1 CPMP  2 CPMP  3 CPMP | 1+2 94.6mo (124.3)  3 72.2mo (88.2) |
| PSEQ | English* | Costa [56] | 2017 | Australia | Pain centre | 1511 | 48.9 (16.10) | 860, 57% | CPMP | NR |
| PSEQ  *(PCS)* | English* | Sleijser [45] | 2019 | Delphi study Europe North America | NR | 36 experts for consensus process | NA | NA | NA | NA |
| PSEQ | Dutch | van der Maas [25] | 2012 | The Netherlands* | Rehabilitation center  Primary care  2 groups | 278* | 1 43.1 (10.5) 2 41.8 ( 9.3) | 1 123, 75%* 2 60, 52.6%* | 1 CPMP 114, 41%* 2 CPMP 164, 59%* | 80.7mo (119.3) |
|  | | | | | | | | | | |
| PIPS | Swedish | Wicksell [20] | 2008 | Sweden | Hospitals  Patient organization | 203 | 45.5 (10.15) | 164, 80.8%* | CWP 62, 30.5%*  CPHP 42, 20.7%*  CPTP 31, 15.3%*  CPMP 25, 12.3%*  Other 43, 21,2% | 10y (6.8) |
| PIPS | Swedish | Wicksell [53] | 2010 | Sweden | Patient organization | 611 | 49 (12.8) | 457, 74.8%* | CPTP | NR |
| PIPS | Dutch | Trompetter [21] | 2014 | The Netherlands | Rehabilitation center | 428 | 43.7 (12.5) | 362, 72.2%* | CPMP 185, 43%*  CWP 113, 26%*  CPTP 61, 14.3%*  CNP 20, 4.7%*  Other 48, 11,2%* | NR |
|  | | | | | | | | | | |
| SF6D12 | English | Luo [57] | 2012 | United Stated of America | National Health Measurement Study Survey | 8913 | 53.9 (NR) | 4662, 52,3%* | CSMP 1355, 15% CPMP 636, 7% | NR |
| SF-12 *(PDI)* | English* | Morris [51] | 2015 | NR | NR | 5965* | NR | NR | CPMP | NR |
| SF-12 | English* | Tawiah [58; 59] | 2018  2019 | Canada | Survey Alberta Retired Teachers Association | 2844 | 68.6 (5.9) | 1559, 54.8%* | CSMP 1041, 36.6%* | 6mo |
| SF-12  *(SCL-90)* | English* | Kroenke [60; 64] | 2019a&b | United States of America | Primary care | 294 | 57.4 (NR) | 37, 12.6%* | CPMP | ≥ 3mo |
|  | | | | | | | | | | |
| SCL-90-R | English* | Kinney [61] | 1991 | United States of America | Rehabilitation center* | 99 | 37 (NR) | 36, 36%* | CPMP | NR |
| SCL-90-R | English* | Bernstein [62] | 1994 | United States of America* | NR | 291 | NR | NR | CPMP | NR |
| SCL-90-R | English* | Peebles [63] | 2001 | Canada* | Rehabilitation center | 158 | 39.6 (11.4) | 100, 63.3%* | CPTP 67, 42.4%* CPMP 91, 57.6%* | 12.1mo (68.2) |
| SCL-90  *(SF-12)* | English* | Kroenke [64] | 2019a&b | United States of America | Primary care | 294 | 57.4 (NR) | 37, 12.6%* | CPMP | ≥ 3mo |

** Interpretation of the reviewers based on ambiguous information. Abbreviations: HADS: Hospital Anxiety and Depression Scale; PCS: Pain Catastrophizing Scale; PDI: Pain Disability Index; PIPS: Psychological Inflexibility in Pain Scale; PSEQ: Pain Self-Efficacy Questionnaire; PSC: Patient-Specific Functional Scale, SCL-90: Symptom Checklist-90; SF-12: 12-Item Short Form Health Survey; SD: Standard Deviation; y: year(s); mo: month(s); wks: weeks; NR: not reported; NA; not applicable; CPMP: chronic primary musculoskeletal pain; CWP: chronic widespread pain; CNP: chronic neuropathic pain; CPHP: chronic pain headache pain; CPTP: chronic post-surgical or post-traumatic pain.*
